# An Extensive Analysis and Comparison of Bone Marrow Aspiration and Bone Marrow Trephine Biopsy at a Tertiary Care Hospital in Jharkhand for Various Hematological and Non-hematological Illnesses

**DOI:** 10.7759/cureus.62661

**Published:** 2024-06-19

**Authors:** Md. Aslam Jawed, Manoj K Paswan, Sunil Kumar Mahto, Satyabrata Patra, Chanchal Ashok, Md. Alimuddin Ansari, Aditi Priya, Abhirup Shome

**Affiliations:** 1 Pathology, Rajendra Institute of Medical Sciences, Ranchi, IND

**Keywords:** bone marrow, simultaneous, comparative evaluation, trephine biopsy, aspirate

## Abstract

Background

Bone marrow examination (BME) is an indispensable diagnostic tool to evaluate various hematological and non-hematological disorders. Bone marrow aspirate cytology and bone marrow trephine biopsy, even though performed simultaneously, are assessed at different points in time due to different processing methods.

Aims and objective

This study aims to assess and compare the role of bone marrow aspiration and trephine biopsy to formulate an effective and rapid method for diagnosing a wide spectrum of various hematological and non-hematological disorders.

Materials and methods

The approach of our study was a hospital-based prospective study conducted on 200 patients over a period of 1 year. The role of bone marrow aspiration and a trephine biopsy is to formulate an effective and rapid method for diagnosing a wide spectrum of hematological and non-hematological disorders.

Results

In our study, a total of 200 cases were studied, of whom 119 patients were male and 81 were female. The most common finding was erythroid hyperplasia, comprising 40 (20%) cases, followed by hypoplastic marrow, comprising 28 (14%) cases. Subsequently, there were 19 (9.5%) cases of acute leukemia, while 15 (7.5%) cases of chronic myeloid leukemia (CML) in the chronic phase were found. In our study, bone marrow aspirate and bone marrow trephine biopsy were found to positively correlate in 137 (68.5%) of the cases.

Conclusion

Bone marrow aspiration alone is sufficient for the diagnosis of megaloblastic anemia and most of the hematological malignancies. Bone marrow trephine biopsy is more appropriate for the detection of disorders of focal marrow involvement such as lymphoproliferative disorders and staging of lymphomas, metastatic cancers, granulomatous lesions, and hypoplastic marrow. However, it is strongly recommended that both procedures should be done simultaneously to ensure maximum diagnostic accuracy.

## Introduction

Bone marrow testing is an essential diagnostic tool for assessing a range of hematological and non-hematological conditions. It is a vital prognostic tool and a crucial part of the follow-up in patients receiving chemotherapy or bone marrow transplants [1,2]. The bone marrow evaluation may either confirm clinically suspected or previously unsuspected diagnoses [3,4]. Bone marrow aspirate cytology (BMA), touch imprint cytology (BMI), and trephine biopsy (BMB) are the three main basic preparations for bone marrow evaluation. BMA is a simple and rapid method for assessing marrow, offering outstanding visualization of cell morphology. Conversely, trephine biopsy delivers more detailed insights into marrow cellularity, trilineage hematopoiesis, and the structural layout of infiltrative marrow disorders [5].

BMA is a commonly performed and safe invasive procedure routinely carried out in hospitals for the diagnosis and evaluation of response to treatment of hematological disorders [6]. In contrast, BMB is a much more painful procedure owing to the bigger bore of the needle and the collection of the material, and its processing takes at least 48-72 hours [5]. Thus, performing trephine biopsies on all patients may not be cost-effective in terms of clinician and laboratory personnel's time, efforts, and patient discomfort. Few studies have analyzed the diagnostic accuracy of bone marrow aspiration relative to trephine biopsy [4,7,8].

In hematology, examining bone marrow is essential for the differential diagnosis of various hematological disorders such as acute leukemia, CLL, hairy cell leukemia, myeloproliferative disorders like polycythemia vera (PV), essential thrombocythemia (ET), primary myelofibrosis (PMF), and chronic myelogenous leukemia (CML), plasma cell dyscrasias like multiple myeloma (MM), staging of lymphomas, and marrow infiltration by foreign cells [9-11].

Bone marrow examination (BME) can precisely diagnose non-hematological conditions like granulomatous lesions, storage diseases, metastatic diseases, various systemic infections, hemophagocytic syndrome, histiocytosis, and leishmaniasis [12-14].

Bone marrow aspirate is the sample of choice to examine nucleated red cells of marrow (M:E ratio). At times, marrow aspirate may be diluted, result in a dry tap, be unsatisfactory, or marrow involvement may be focal, such as in granulomas, metastatic deposits, focal myeloma, Hodgkin’s disease, and associated fibrosis. In such cases, a trephine biopsy is essential to arrive at a diagnosis. The diagnostic accuracy of the two diagnostic modalities differs for different conditions [15].

Evaluating both marrow aspirate and trephine biopsy together enables a more comprehensive assessment of the marrow, which might not be possible when using either method alone [5].

Although marrow aspirate and biopsy are safe invasive procedures with a low risk of bleeding, they are considered quite safe even in cases of severe thrombocytopenia [16].

## Materials and methods

Study design and duration

A hospital-based prospective study was conducted on 200 patients over a period of one year from 2022 to 2023 at the Department of Pathology, RIMS, after obtaining ethical approval (Approval No. 122/IEC/RIMS).

Inclusion criteria

The study subjects consisted of clinically suspected cases of either hematological or non-hematological disorders in the age group between 2 and 70 years attending the Department of Pathology, RIMS Ranchi, for bone marrow evaluation.

Exclusion criteria

Patients with bleeding or coagulation disorders, ages less than two years or more than 70 years, and terminally ill patients were excluded. 

Methodology

The study was conducted in the Department of Pathology, Rajendra Institute of Medical Sciences, Ranchi, after obtaining informed consent. The demographic profile of all study patients and a detailed clinical history including presenting complaints, dietary history, and drug history were recorded. History of radiotherapy and chemotherapy was also included. Relevant clinical details and results of previous investigations such as complete blood count (CBC), peripheral blood smear (PBS), reticulocyte count, immature reticulocyte fraction (IRF), and coagulation profile were reviewed. The study comprised 200 patients in total when aspiration and trephine biopsy were performed concurrently.

Bone marrow aspirate and trephine biopsy

BMA was performed using Salah’s needle, and 0.25 to 0.5 mL of aspirate was withdrawn with a 20 mL plastic syringe from the posterior superior iliac spine (PSIS). The needle and syringe were withdrawn together, and the marrow was poured onto slides placed at a 30-degree angle to drain off the blood present in the marrow. Smears of bone marrow were made quickly to avoid clotting and stained with Leishman’s stain. Good marrow smears contain marrow particles as well as a trail of particles. After that, a trephine biopsy was performed using Jamshidi’s needle through the same incision, approximately 0.5-1 cm away from the site of aspiration to avoid a hemorrhagic biopsy. A peripheral blood film was also prepared simultaneously. PBS and BMA were stained with Leishman’s stain.

In our study, optimal processing of bone marrow trephine biopsy was conducted as per the Hammersmith protocol. Trephine biopsies were fixed in the Acetic Acid-Zinc-Formalin (AZF) fixative fluid overnight for 20-24 hours. Following this, they were washed in distilled water for 30 minutes and subjected to decalcification in 10% formic acid and 5% formaldehyde solution for three to five hours. Once decalcification began, the biopsy was removed and placed in water for 30 minutes. Subsequently, the length of the biopsy was recorded. It was then processed routinely in an automated tissue processor, embedded in paraffin, sectioned into 2-3 µm thick slices, and stained with hematoxylin and eosin (H&E stain).

The reports of both aspirate and biopsy for each case were reviewed and compared. The reasons for inconclusive reports were also recorded.

Statistical analysis

The results were statistically analyzed using IBM SPSS (IBM Corp., Armonk, New York) and Excel. Categorical variables such as age, gender, and cellularity were expressed with their mean. BMA and BMB were considered gold-standard investigations for the diagnosis and monitoring of many hematological and non-hematological disorders. Positive correlations and results were compared.

## Results

Age and gender variation

In our study, a total of 200 cases were examined. Of these, 119 patients were male and 81 were female. The mean age of the study population was 30.91±1.92 years. Most of the study population was between two and 20 years of age. However, while the maximum number of male cases was found in this age group, the number of female cases was higher in the 21-40 years age group (Table 1). 

**Table 1:** Demographic profile of the study population

Variables		Cases	Male	Female
		(n=200)		
Study population		200	119	81
Age groups	02-20	76	51	25
	21-40	62	30	32
	41-60	47	27	20
	61-70	15	11	4

The total number of cases reviewed was 200, where both BMA and BMB were performed together. The gender-wise distribution of bone marrow cellularity is detailed in Table 2, and the distribution of bone marrow cellularity revealed some variations among the participants. Normocellular marrow was observed in 62 (31%) cases, with a male predominance of 38 cases compared to 24 females. Hypocellular marrow was noted in 33 (16.5%) cases, with 21 males and 12 females. The majority of the cases, 105 (52.5%), exhibited hypercellular marrow, showing a higher prevalence in males (60 cases) compared to females (45 cases).

**Table 2:** Gender-wise distribution of bone marrow cellularity

Variable	Cases	Male	Female	Percentage
	N=200			
Normocellular	62	38	24	31%
Hypocellular	33	21	12	16.50%
Hypercellular	105	60	45	52.5%

Normal study results were found in 27 (13.5%) cases, more in males (n=19) than females (n=8). Erythroid hyperplasia was the most common diagnosis, accounting for 40 (20%) cases. Reactive marrow was observed in 11 (5.5%) cases and megaloblastic marrow in 16 (8%) cases. Hypoplastic marrow was noted in 28 (14%) cases, predominantly in males (19). Acute leukemia was diagnosed in 19 cases (9.5%), with AML and ALL each comprising three (1.5%) cases. CML-CP was found in 15 (7.5%) cases and CML-BL in one (0.5%) case. Other notable diagnoses included ITP and hypersplenism, each with six (3%) cases, and MM with seven (3.5%) cases. Various other conditions such as metastasis, lymphoma, Gaucher’s disease, MDS, PV, and ET each accounted for a small fraction. Overall, 119 cases involved males and 81 involved females. This gender-wise distribution for final diagnoses is presented in Table 3 and Table 4.

**Table 3:** Gender-wise distribution of final diagnosis AML, acute myeloid leukemia; ALL, acute lymphocytic leukemia; CML-CP, chronic myeloid leukemia in chronic phase; CML-BP, chronic myeloid leukemia in blast phase; MDS, myelodysplastic syndrome; PV, polycythemia vera; ET, essential thrombocythemia; ITP, immune thrombocytopenia; MM, multiple myeloma; LD, Leishman-Donovan

Variable	Male	Female	Total	Percentage
Normal study	19	8	27	13.50%
Erythroid hyperplasia	23	17	40	20%
Reactive marrow	4	7	11	5.50%
Megaloblastic marrow	7	9	16	8%
Hypoplastic marrow	19	9	28	14%
Acute leukemia	13	6	19	9.50%
AML	2	1	3	1.50%
ALL	2	1	3	1.50%
CML-CP	8	7	15	7.50%
CML-BL	1	0	1	0.50%
ITP	2	4	6	3%
Hypersplenism	2	4	6	3%
MM	5	2	7	3.50%
Metastasis	1	1	2	1%
Lymphoma	1	1	2	1%
Gaucher’s disease	1	0	1	0.50%
MDS	2	0	2	1%
PV	1	0	1	0.50%
LD bodies	1	0	1	0.50%
ET	1	0	1	0.50%
Myeloid hyperplasia	4	4	8	4%
Total	119	81	200	100%

**Table 4:** Age group-wise distribution of final diagnosis AML, acute myeloid leukemia; ALL, acute lymphocytic leukemia; CML-CP, chronic myeloid leukemia in chronic phase; CML-BP, chronic myeloid leukemia in blast phase; MDS, myelodysplastic syndrome; PV, polycythemia vera; ET, essential thrombocythemia; ITP, immune thrombocytopenia; MM, multiple myeloma; LD, Leishman-Donovan

Variable	02-20	21-40	41-60	61-70	Total
Normal study	10	7	8	2	27
Erythroid hyperplasia	16	13	8	3	40
Reactive marrow	2	8	1	0	11
Megaloblastic marrow	4	7	4	1	16
Hypoplastic marrow	13	7	5	3	28
Acute leukemia	13	3	2	1	19
AML	1	1	1	0	3
ALL	3	0	0	0	3
CML-CP	1	8	5	1	15
CML-BL	0	0	1	0	1
ITP	2	0	3	1	6
Hypersplenism	4	2	0	0	6
MM	0	0	5	2	7
Metastasis	0	0	2	0	2
Lymphoma	1	1	0	0	2
Gaucher’s disease	0	1	0	0	1
MDS	2	0	0	0	2
PV	0	0	1	0	1
LD bodies	0	1	0	0	1
ET	0	0	0	1	1
Myeloid hyperplasia	4	3	1	0	8
Total	76	62	47	15	200

Out of the 200 cases studied, there was a positive correlation in 137 (68.5%) cases between marrow aspiration and trephine biopsy. The diagnoses included normal study for 17 (12.40%) cases, erythroid hyperplasia in 24 (17.50%) cases, reactive marrow in 19 (13.80%) cases, megaloblastic marrow in 14 (10.20%) cases, hypoplastic marrow in 12 (8.75%) cases, acute leukemia in 12 cases (8.75%), with AML in three (2.20%) cases, ALL in three (2.20%) cases, CML-CP in seven (5.10%) cases, CML-BP in one (0.70%) case, hypersplenism in four (2.90%) cases, MM for five (3.60%) cases, metastasis in two (1.40%) cases, granulomatous disease for two (1.40%) cases, Gaucher’s disease for one (0.70%) case, MDS for two (1.40%), PV in one (0.70%) case, ET in two (1.40%) cases, Leishman-Donovan (LD) bodies in two (1.40%) cases, and ITP for four (2.90%) cases. These disease-specific details are provided in Table 5.

**Table 5:** Positive correlation between bone marrow aspiration and bone marrow trephine biopsy AML, acute myeloid leukemia; ALL, acute lymphocytic leukemia; CML-CP, chronic myeloid leukemia in chronic phase; CML-BP, chronic myeloid leukemia in blast phase; MDS, myelodysplastic syndrome; PV, polycythemia vera; ET, essential thrombocythemia; ITP, immune thrombocytopenia; MM, multiple myeloma; LD, Leishman-Donovan

S. No.	Diagnosis	No. of cases	Percentage
1	Normal study	17	12.40%
2	Erythroid hyperplasia	24	17.50%
3	Reactive marrow	19	13.80%
4	Megaloblastic marrow	14	10.20%
5	Hypoplastic marrow	12	8.75%
6	Acute leukemia	12	8.75%
7	AML	3	2.20%
8	ALL	3	2.20%
9	CML-CP	7	5.10%
10	CML-BP	1	0.70%
11	Hypersplenism	4	2.90%
12	MM	5	3.60%
13	Metastasis	2	1.40%
14	Granulomatous disease	2	1.40%
15	Gaucher’s disease	1	0.70%
16	MDS	2	1.40%
17	PV	1	0.70%
18	ET	2	1.40%
19	LD bodies	2	1.40%
20	ITP	4	2.90%

**Figure 1:**
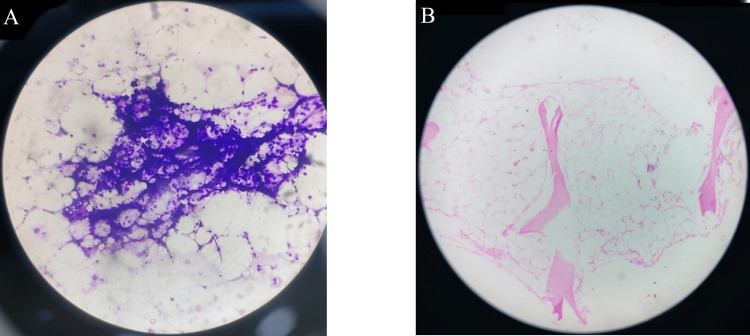
Photomicrograph of (A) bone marrow aspiration and (B) bone marrow trephine biopsy of a case of aplastic anemia Hypocellular bone marrow with increased fat, and no megakaryocytic, erythroid, or myeloid precursors seen.

**Figure 2:**
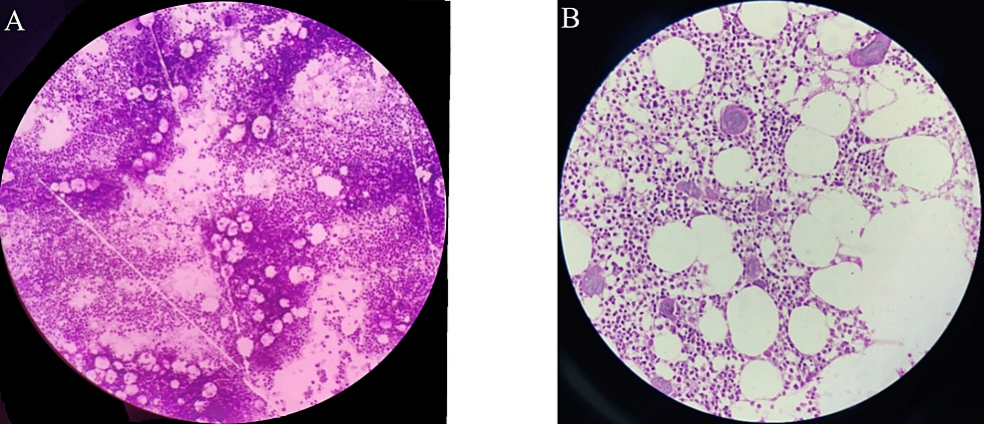
Photomicrograph of (A) bone marrow aspiration and (B) bone marrow trephine biopsy of a case of ET, showing 100% correlation (A) Marrow aspiration reveals focal clustering of megakaryocytes. (B) Megakaryocytic hyperplasia with loose clusters, predominantly giant forms, is seen on trephine biopsy. ET, essential thrombocythemia

**Figure 3:**
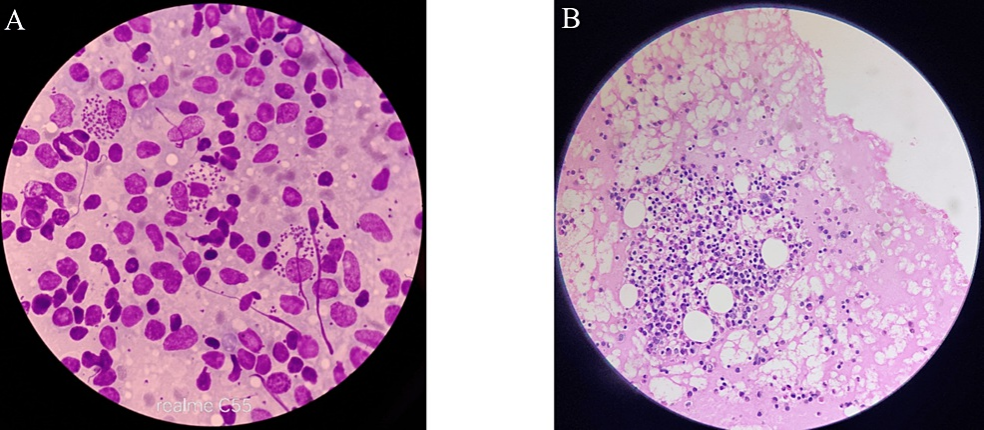
Photomicrograph of (A) bone marrow aspiration and (B) bone marrow trephine biopsy of a case of kala-azar LD bodies (cluster of LD bodies with nucleus and kinetoplast) (A) Intracellular and extracellular Leishman-Donovan (LD) bodies are seen, with distinct kinetoplasts. (B) The marrow is hypercellular with increased plasma cells. Macrophages show multiple dot-like inclusion bodies (LD bodies). LD, Leishman-Donovan

**Figure 4:**
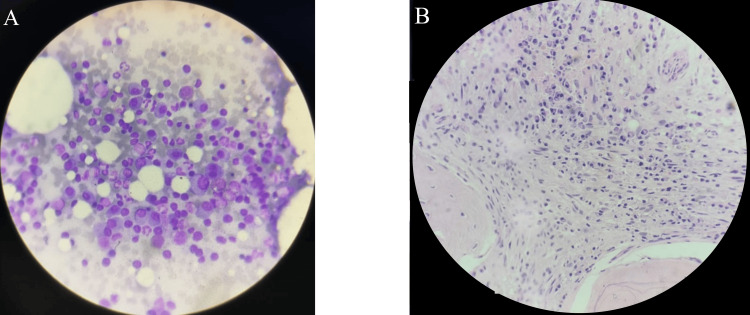
Photomicrograph of (A) bone marrow aspiration and (B) bone marrow trephine biopsy of a case of MM (A) Sheets of plasma cells with eccentric nuclei; few are binucleated or trinucleated. (B) Numerous plasma cells of intermediate differentiation are seen in a diffuse pattern. MM, multiple myeloma

**Figure 5:**
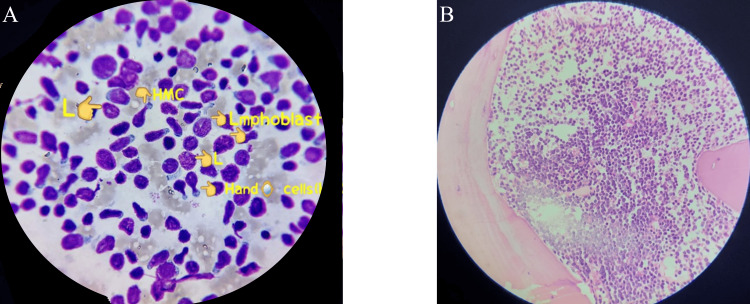
Photomicrograph of (A) bone marrow aspiration and (B) bone marrow trephine biopsy of a case of acute leukemia (A) Hypercellular marrow with blasts of varying sizes, prominent one to two nucleoli, and Hand-Mirror cells are seen. (B) The marrow is hypercellular, with a predominance of immature blasts.

The photomicrographs of the bone marrow aspiration and the bone marrow trephine biopsy are shown in Figures 1-5. Figure 1 shows hypocellular bone marrow with increased fat, and no megakaryocytic, erythroid, or myeloid precursors. 

Figure 2 shows marrow aspiration revealing focal clustering of megakaryocytes. Megakaryocytic hyperplasia with loose clusters, predominantly giant forms, is seen on BMB with an increased number of diffusely scattered, mature, large multilobated megakaryocytes.

Figure 3 shows intracellular and extracellular LD bodies with distinct kinetoplasts. The marrow is hypercellular with increased plasma cells, and macrophages display multiple dot-like inclusion bodies (LD bodies).

Figure 4 shows sheets of plasma cells with eccentric nuclei, with a few being binucleated or trinucleated. Additionally, numerous plasma cells of intermediate differentiation are seen in a diffuse pattern, which is characteristic of MM. Pleomorphism of the plasma cells is also observed.

Figure 5 shows the bone marrow slide for acute leukemia. It reveals a hypercellular marrow with blasts of varying sizes, prominent one to two nucleoli, and Hand-Mirror cells. Additionally, the marrow is hypercellular with a predominance of immature blasts, which is indicative of acute leukemia.

Table 6 illustrates the diagnoses made based on bone marrow trephine biopsy alone. Of these, tuberculous granuloma was the most frequent diagnosis, accounting for six (26.10%) cases. Leukemia was identified in five (21.70%) cases, while MM and chronic myeloproliferative disorders were each found in three (13.04%) cases. Hodgkin's disease and metastasis were diagnosed in two (8.70%) cases each. Additionally, Gaucher’s disease and ITP were each observed in one (4.30%) case.

**Table 6:** Diagnosis on bone marrow biopsy alone MM, multiple myeloma

S. No	Diagnosis	No. of cases	Percentage
1	Tuberculous granuloma	6	26.10%
2	Hodgkin’s disease	2	8.70%
3	MM	3	13.04%
4	Leukemia	5	21.70%
5	Chronic myeloproliferative disorders	3	13.04%
6	Gaucher’s disease	1	4.30%
7	Metastasis	2	8.70%
8	ITP	1	4.30%

**Figure 6:**
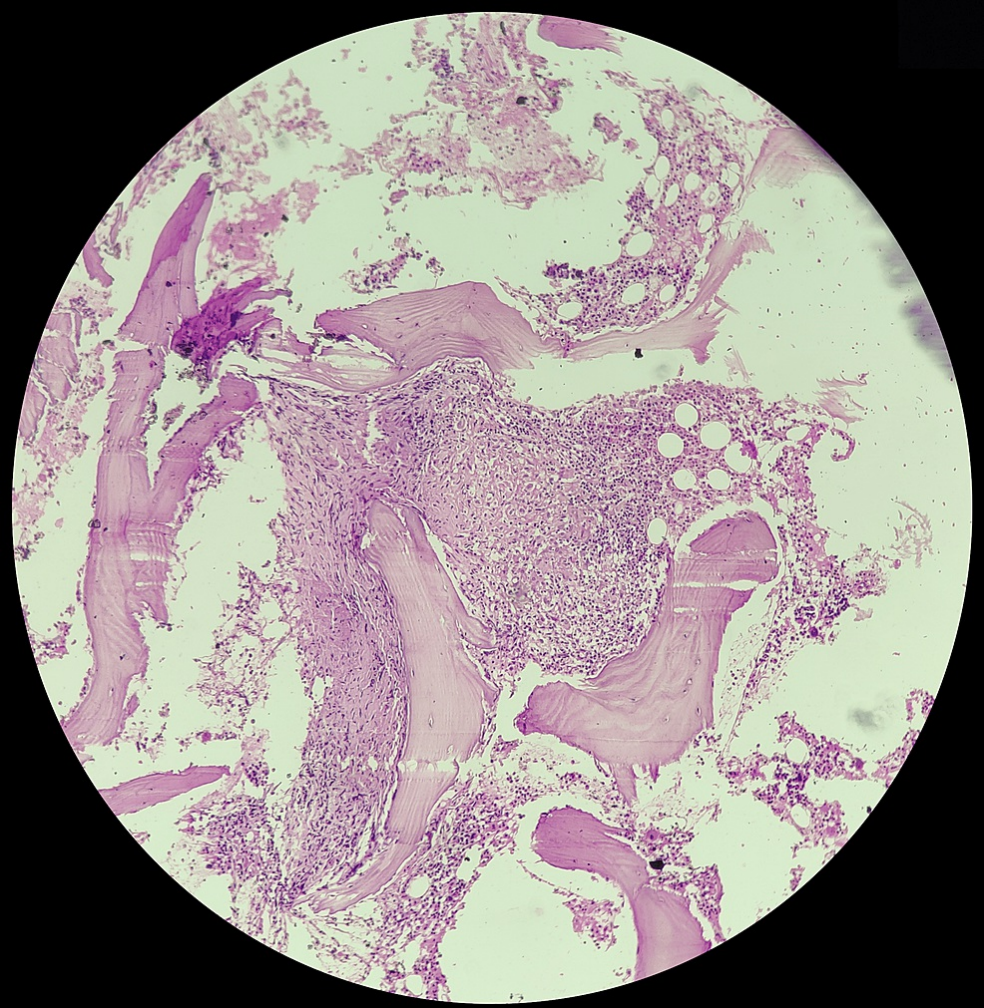
Photomicrograph of tuberculous granuloma in trephine biopsy The cellular marrow shows granulomas composed of epithelioid cells, suggestive of tuberculosis.

Figure 6 shows the microscopic picture of a tuberculous granuloma. The cellular marrow reveals granulomas composed of epithelioid cells, which are suggestive of tuberculosis, and is characterized by a collection of epithelioid macrophages, often surrounded by lymphocytes, and may also contain multinucleated giant cells. 

**Figure 7:**
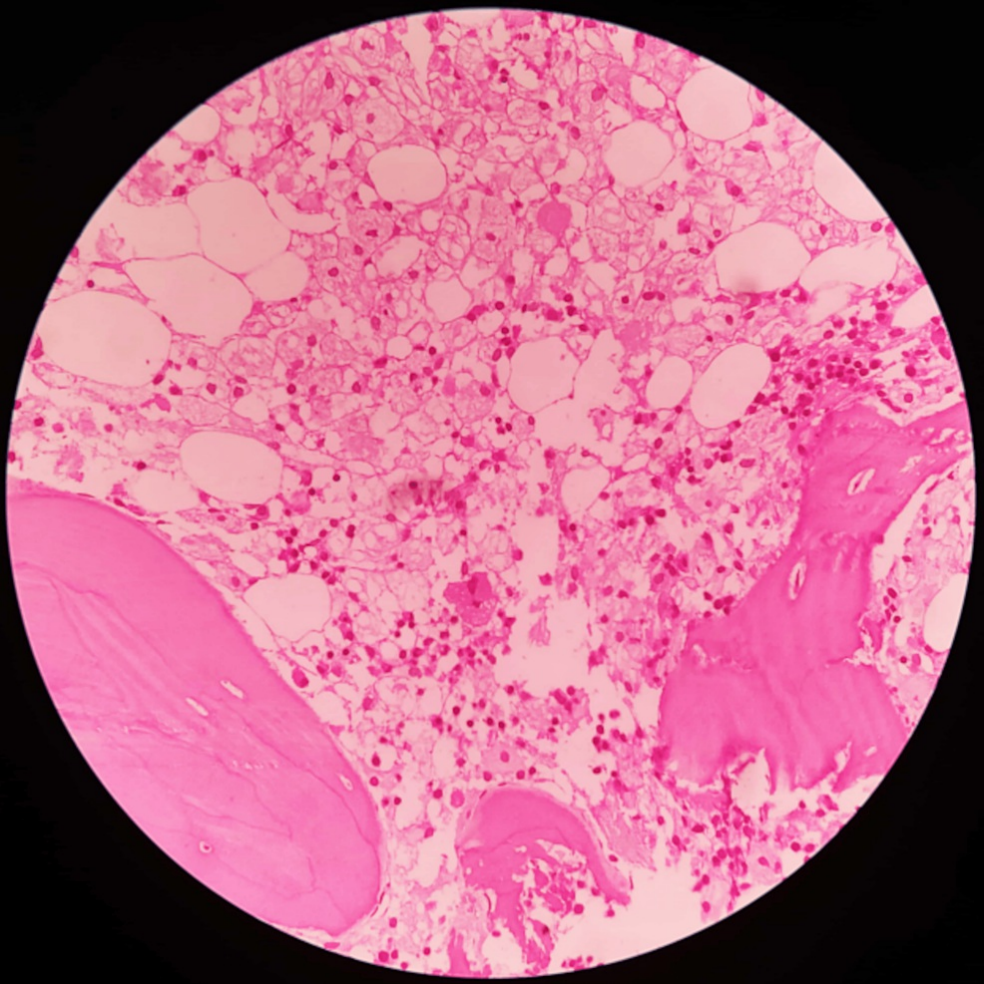
Photomicrograph of Gaucher’s disease in trephine biopsy Sheets of Gaucher's cells are seen with small, eccentric nuclei and abundant cytoplasm with a characteristic fibrillar appearance, resembling wrinkled or crumpled tissue paper.

Figure 7 shows the microscopic picture of Gaucher's disease in BMB. The photomicrograph reveals sheets of Gaucher's cells with small, eccentric nuclei and abundant cytoplasm with a characteristic fibrillar appearance, resembling wrinkled or crumpled tissue paper.

**Figure 8:**
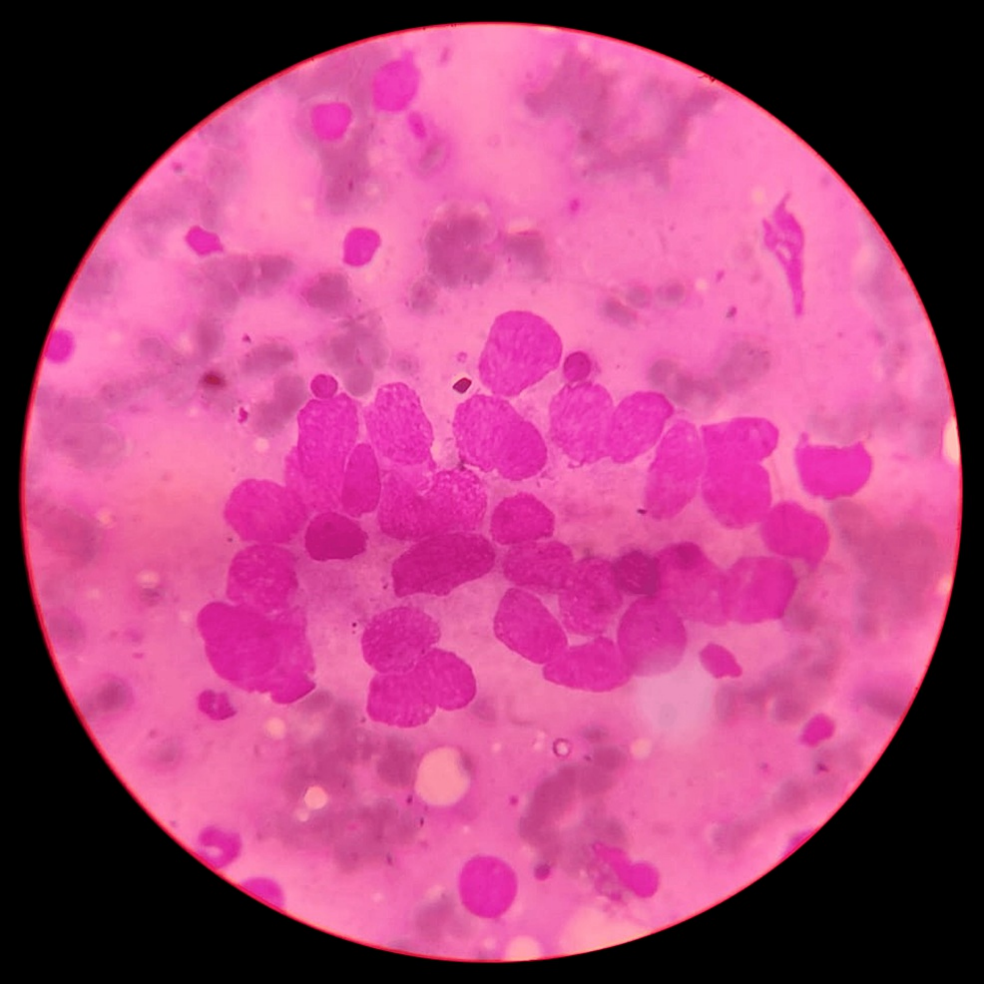
Photomicrograph of metastatic deposits in bone marrow aspirate BMA reveals foreign cells arranged in an acinar pattern, with some scattered individually and in clusters. Malignant cells within the clusters exhibit pleomorphism.

Figure 8 depicts a photomicrograph of metastatic deposits on bone marrow aspirate. The aspirate shows foreign cells arranged in an acinar pattern, with a few in discrete patterns and also in clusters. The smear shows metastatic malignant cells with hyperchromatic nuclei and prominent nucleoli.

There were 23 (11.5%) cases where the diagnosis was possible only with BMB, as BMA was non-contributory. There was a single case diagnosed as metastasis to the marrow by BMA, where BMB showed an infarcted marrow (Figure 8). Cases where a definitive opinion could not be given, either in BMA or BMB, comprised 40 out of 200 (20%). The findings ranged from a hypercellular or reactive trephine biopsy to an insufficient marrow aspiration. Marrow aspiration appeared normal in certain cases, but trephine biopsy revealed no marrow gaps.

In our investigation at a tertiary center in Jharkhand, we discovered that cases identified as erythroid hyperplasia (24 (17.5%)) and reactive marrow (19 (13.8%)) had the highest correlation rates. In cases diagnosed with erythroid hyperplasia, whether the patient was macro- or micronormoblastic, they underwent additional testing primarily for anemia. Perl's stain for iron was examined in these cases, biochemical parameters were considered, and an impression was made. Both marrow aspiration and trephine biopsy were present in cases with megaloblastic anemia. The megaloblasts in marrow aspiration were easily identified; however, it is well known that the megaloblasts in trephine biopsy resembled leukemic blasts.

Hematological malignancies, including MM, exhibited a strong positive correlation. Five of the eight (62.5%) instances diagnosed had positive correlations in marrow aspiration and trephine biopsy. In 12 out of 17 (70.5%) instances, leukemia exhibited a positive correlation; those with a BMB diagnosis alone had a hypocellular BMA. The correlation with ET was two out of two (100%). Two out of four (50%) of the non-hematological malignancies (those that metastasized to the bone marrow) had positive correlations in both marrow aspiration and trephine biopsy. Based on the correlation results we found, the data can be tabulated as shown in Table 7.

**Table 7:** Correlation rates between bone marrow aspiration and bone marrow biopsy ET, essential thrombocythemia

Diagnostic category	Cases (n)	Cases (%)	Correlation rate	Remarks
BMB alone diagnostic	23	11.50%	N/A	BMA non-contributory
BMA diagnosed metastasis	1	0.50%	N/A	BMB showed infarcted marrow
Indeterminate (BMA or BMB)	40	20%	N/A	Hypercellular/reactive trephine biopsy or insufficient marrow aspiration
Normal BMA but abnormal BMB	N/A	N/A	N/A	Trephine biopsy revealed no marrow gaps
Erythroid hyperplasia	35	17.50%	High correlation	Diagnosed for anemia, additional testing (Perl's stain for iron, biochemical parameters)
Reactive marrow	27.6	13.80%	High correlation	N/A
Megaloblastic anemia	N/A	N/A	N/A	Megaloblasts easily identified in BMA but resembled leukemic blasts in trephine biopsy
Hematological malignancies (e.g., myeloma)	8	N/A	62.5% (5/8)	Strong positive correlation
Leukemia	17	N/A	70.5% (12/17)	Hypocellular BMA in cases diagnosed by BMB alone
ET	N/A	N/A	100%	N/A
Non-hematological malignancies	4	N/A	50% (2/4)	Metastasized to bone marrow

## Discussion

BMA and BMB are crucial diagnostic tools for identifying various hematological and non-hematological disorders. These procedures are also valuable for follow-up in patients undergoing chemotherapy and other medical treatments, including bone marrow transplantation [1,2]. It is well-established that BMA and BMB are complementary procedures. Nowadays, it is customary to collect both specimens simultaneously, preferably from the same location [17].

Our study at the tertiary center of Jharkhand (RIMS) was conducted for a comparative review of these procedures to understand their complementary roles and the benefits and drawbacks of performing both of them simultaneously. In our study, we found a positive correlation between BMA and BMB in 137 (68.5%) cases. The strongest positive correlation was observed in cases with erythroid hyperplasia (24 (17.5%)) and reactive marrow (19 (13.8%)). Our findings were consistent with the study done by Pampa Ch Toi et al. [1].

In our study, megaloblastic anemia was the most common cause of pancytopenia and the most common finding in BMA, reflecting the higher prevalence of nutritional deficiency due to poverty, which is prevalent in most of the underserved parts of Jharkhand. The most common age group with megaloblastic marrow was 21-40 years, which is consistent with a similar study, done by Jha et al. [18]. In one case of megaloblastic anemia, both BMA and BMB were performed concurrently due to the patient’s pancytopenia. Although BMA alone is usually sufficient for diagnosing clinically suspected megaloblastic anemia, the simultaneous use of BMB revealed numerous blasts, which could have been misinterpreted as leukemia. However, the characteristics of megaloblasts observed in BMA were crucial for the correct diagnosis [19]. Thus, while BMB is not typically used for diagnosing megaloblastic anemia, BMA proved to be essential.

For MM, we observed a positive correlation between marrow aspiration and trephine biopsy in five out of eight (62.5%) cases. Although MM can often be diagnosed using marrow aspiration alone, there are instances where plasma cells are dispersed in the aspirate. In such cases, trephine biopsy complements marrow aspiration by identifying compact masses of plasma cells without stroma, as demonstrated by Sabharwal et al. [20]. Our findings were consistent with those of Charles et al., who also found MM in concurrent BMA and BMB [21].

For acute leukemia, we observed a positive correlation in 12 out of 17 (70.5%) cases. The sensitivity of BMA as a diagnostic procedure depends on the disease being evaluated. Diseases with diffuse involvement of bone marrow are diagnosed with adequate sensitivity by BMA alone. A high concordance rate was obtained in a considerable proportion of cases of acute leukemia, and BMA alone may suffice as a diagnostic procedure in most cases. However, a marrow completely replaced by blasts or immature myeloid precursors is often difficult to aspirate and results in a dry tap. Thus, BMB is essential in all such cases. The results of another study also found that acute leukemias were diagnosed on aspiration alone; trephine biopsy provided additional useful information [22].

In our study, all patients with Hodgkin’s disease were diagnosed using trephine biopsy alone. One patient clinically suspected of tuberculosis was found to have Hodgkin's disease through trephine biopsy, while BMA showed reactive marrow. These findings align with Moid et al., who reported a 95% diagnosis of Hodgkin's disease using trephine biopsy alone, and Howell et al., who noted that marrow aspiration did not significantly aid in detecting marrow involvement in Hodgkin's disease [23,24]. Similarly, Sharma et al. reported diagnosing Hodgkin's disease solely through trephine biopsy [25].

We found that six out of eight (75%) of granulomatous lesions in the bone marrow were detected exclusively by trephine biopsy, with most cases being tuberculous granulomas. Granulomatous changes can be specific or nonspecific [26]. Although the AFB stain was not positive in all instances, the clinical history, presentation, and other studies confirmed the diagnosis of tuberculous granuloma. Thus, trephine biopsy is a superior method for detecting granulomas in the marrow.

For leishmaniasis, over 90% of kala azar cases in India are reported from Bihar and Jharkhand. At our tertiary center, patients presented with complaints of fever, weight loss, and hepatosplenomegaly. The probable reason may be delayed diagnosis due to the late presentation of cases from remote areas in the study or association with other infections such as malaria, TB, or enteric fever. In our study, two (1.4%) cases showed LD bodies reported on BMA and BMB. Similar findings were seen in a study done by Santra et al., but the maximum number of cases (14%) was seen in a study done by Khodke et al. [27,28]. This highlights the importance of vigilant BME for the search of LD bodies and observation of associated bone marrow features even if the clinical suspicion is low. In our study, one case (0.7%) of Gaucher's disease, two (1.4%) cases of metastatic infiltration, and four (2.9%) cases of ITP were reported on BME. Similar results were also found by Khan MI et al. [29].

Though these two techniques are commonly utilized, BMB alone with a good impression smear suffices in cases where Hodgkin's disease or tuberculosis is suspected. Our study also focused on cases where a definitive opinion could not be provided by either BMA or BMB, accounting for 40 out of 200 (20%) cases. The difficulty often arose from insufficient or diluted samples. In some instances, BMA appeared normal, whereas BMB revealed either hypercellular or hypocellular marrow. Despite these challenges, the benefits of performing both procedures concurrently outweigh the drawbacks, as both BMA and BMB are painful. Therefore, obtaining a good sample while considering patient comfort is crucial.

When performing both procedures simultaneously, the two-needle technique is recommended. This involves moving the needle to a neighboring spot after one procedure to extract the most material. Recently, we have begun performing imprint smears on all trephine biopsies at our tertiary facility, which has proven highly valuable for studying cell morphology.

We follow the Hammersmith protocol and the two-needle approach for BMA and BMB at our tertiary center. Our observations suggest that the two-needle technique is beneficial [17]. By employing this technique, both procedures can be completed simultaneously, potentially providing valuable diagnostic data.

Limitations

The limitations of this study include it being a descriptive single-institution study in Jharkhand, potentially limiting generalizability. Additionally, the lower sample size may restrict the strength of the conclusions inferred. Moreover, the lack of access to cytogenetic techniques and molecular studies hindered further classification and confirmation of certain diagnoses. These constraints emphasize the importance of larger, multi-center studies with comprehensive diagnostic tools.

## Conclusions

Marrow aspiration and trephine biopsy are frequently utilized diagnostic procedures that work in tandem with one another. Based on our experience, we believe that both procedures can be performed concurrently for diagnostic purposes. BMA provides a better image of cell morphology, while BMB offers a good picture of the topography and architecture of the marrow.

We discovered that BMB was particularly helpful in diagnosing non-hematological malignancies, metastases, tuberculous granuloma, and Hodgkin's disease. When performing marrow aspiration and trephine biopsy simultaneously, proper technique should be maintained to yield the maximum amount of material and minimize patient discomfort by avoiding the need to repeat the procedure due to insufficient material. Marrow aspiration and trephine biopsy are helpful in clinching an early and correct diagnosis of hematological and non-hematological illnesses, thereby aiding in the provision of personalized treatment for the patient.
